# Oncoprotein HBXIP promotes tumorigenesis through MAPK/ERK pathway activation in non-small cell lung cancer

**DOI:** 10.20892/j.issn.2095-3941.2020.0098

**Published:** 2021-02-15

**Authors:** Jun Zhang, Bei Sun, Xianhui Ruan, Xiukun Hou, Jingtai Zhi, Xiangrui Meng, Xiangqian Zheng, Ming Gao

**Affiliations:** 1 Department of Breast Cancer, Key Laboratory of Breast Cancer Prevention and Therapy, Tianjin Medical University Cancer Institute and Hospital, National Clinical Research Center for Cancer, Key Laboratory of Cancer Prevention and Therapy, Tianjin, Tianjin’s Clinical Research Center for Cancer, Tianjin 300060, China; 2 Department of Outpatient Office, Tianjin Medical University Cancer Institute and Hospital, National Clinical Research Center for Cancer, Key Laboratory of Cancer Prevention and Therapy, Tianjin, Tianjin’s Clinical Research Center for Cancer, Tianjin 300060, China; 3 Department of Thyroid and Neck Tumor, Tianjin Medical University Cancer Institute and Hospital, National Clinical Research Center for Cancer, Key Laboratory of Cancer Prevention and Therapy, Tianjin, Tianjin’s Clinical Research Center for Cancer, Tianjin 300060, China; 4 Department of Lymphoma, Tianjin Medical University Cancer Institute and Hospital, National Clinical Research Center for Cancer, Key Laboratory of Cancer Prevention and Therapy, Tianjin, Tianjin’s Clinical Research Center for Cancer, Tianjin 300060, China

**Keywords:** HBXIP, non-small cell lung cancer, MEK1, tumor progression

## Abstract

**Objective:**

The oncoprotein, hepatitis B X-interacting protein (HBXIP), has been reported to play an important role in human malignancies. However, its functions in non-small cell lung cancer (NSCLC) are poorly understood. The goal of the present study was to identify the role of HBXIP in the regulation of NSCLC development.

**Methods:**

The level of HBXIP expression in NSCLC tissue was assessed by immunohistochemical and Western blot analyses, and its relationships with clinicopathological features and outcomes were statistically evaluated. The effects of HBXIP on NSCLC cell progression were assessed through cell viability, colony formation, and flow cytometry analyses *in vitro*. The mechanism by which HBXIP regulated the MAPK pathway was studied by Western blot, immunofluorescence, and immunoprecipitation assays. In addition, *in vivo* experiments were performed to evaluate the progression of NSCLC and ERK signaling pathway activation after HBXIP knockdown.

**Results:**

HBXIP was overexpressed in human NSCLC and was correlated with the invasiveness of NSCLC. The high expression of HBXIP in NSCLC was significantly correlated with gender (*P* = 0.033), N stage (*P* = 0.002), and tumor-node-metastasis stage (*P* = 0.008). *In vitro* experiments using an NSCLC cell line revealed that HBXIP knockdown resulted in the suppression of cell proliferation and colony formation, which was consistent with the enhanced cell cycle arrest in G1 phase. The results of a mechanistic investigation suggested that binding of HBXIP to MEK1 protein promoted MAPK/ERK signaling pathway activation in NSCLC by preventing the proteasome-mediated degradation of MEK1. In addition, the results obtained using *in vivo* subcutaneous tumor xenografts confirmed that HBXIP deficiency decreased MEK1 protein levels and NSCLC tumor growth.

**Conclusions:**

Taken together, our results showed that the HBXIP-MEK interaction promoted oncogenesis *via* the MAPK/ERK pathway, which may serve as a novel therapeutic target for cancers in which MAPK/ERK signaling is a dominant feature.

## Introduction

Lung cancer is the leading cause of cancer-related deaths worldwide, of which non-small cell lung cancer (NSCLC) is the primary subtype, accounting for 80% of all new lung cancer cases in men and women^1–3^. Surgery, chemotherapy, and radiotherapy are common treatments for NSCLC, but despite improvements in multimodal treatment strategies made in previous decades, the prognoses of NSCLC patients remain poor, with a 5-year survival of less than 15% after the initial diagnosis^4–6^. Recent studies have shown that genetic factors may be the major cause of NSCLC^7^. Recently, several molecular signaling pathways, involving mutations in the MAPK and PI3K/AKT pathways, including RAS and EGFR mutations, have been reported to contribute to NSCLC progression^8,9^. Nevertheless, the specific mechanisms by which these mutations contribute to NSCLC remain largely unknown.

Hepatitis B X-interacting protein (HBXIP, also known as LAMTOR5) encodes a 91 amino acid protein that is conserved among mammals. It was originally identified by its interaction with the C-terminus of HBx (hepatitis B virus x)^10^. A growing body of evidence from the past decade suggests that HBXIP is a novel tumor promoter in different types of cancers, including liver, breast, bladder, cervix, and ovary cancers^11–15^. The regulation of HBXIP in cancer has been shown to have a broad range of effects. For example, HBXIP can suppress apoptosis through survivin/caspase9 in hepatoma cells^16^ and regulate centrosome duplication in HeLa cells^17,18^. HBXIP can also promote the progression of breast and liver cancers *via* the MAPK/ERK and PI3K/Akt pathways. Recent studies have shown that HBXIP acts as a novel coactivator of transcription factors, such as E2F1 and TFIID, promoting the proliferation and metastases of breast cancer^19–22^. Moreover, HBXIP is essential for the lysosomal localization of Rag GTPases and mTORC1 as well as the subsequent activation of mTORC1 in response to amino acid signaling^23^.

In the present study, we therefore examined the expression of HBXIP in NSCLC and its association with clinicopathological parameters. The role of HBXIP in the regulation of NSCLC development was also investigated.

## Materials and methods

### Tissue specimens and immunohistochemistry

Forty lung adjacent samples, 40 inflammatory pseudotumor samples, and 159 NSCLC tumor samples used for microarray analysis were obtained from patients registered at the Tianjin Medical University Cancer Institute and Hospital (Tianjin, China). This patient study was approved by the Ethics Committee of Tianjin Medical University (approval number: bc2020063), and all tumors were assayed in duplicate (1.5 mm tissue cores). Samples for immunohistochemistry (IHC) analysis were fixed with 4% formalin and embedded in paraffin. IHC was performed according to standard procedures. Briefly, tissue sections were incubated with an anti-human HBXIP antibody at a 1:400 dilution (#ABE807; Millipore, Hayward, CA, USA) followed by visualization with a 3,3′-diaminobenzidine substrate kit (MaiXin Bio, Shenzhen, China) and imaging with a phase contrast light microscope (Leica, Wetzlar, Germany). HBXIP protein intensity was assessed according to previously reported methods^24^. Staining intensity was scored as follows: 0 (–), 1 (+), 2 (++), and 3 (+++). The degree of staining was categorized as 0 (0% staining), 1 (1%–30% staining), 2 (31%–60% staining), 3 (61%–80% staining), and 4 (81%–100% staining). The final score was determined by multiplying the staining intensity score by the degree of staining, ranging from 0–12. Samples with a final score < 3 were considered to have low expression, while those with a score of 4–12 were considered to have high expression.

### Cell culture and stable cell line establishment

The NSCLC cell lines, A549 and H1299, and HEK293T cells were purchased from the American Type Culture Collection (ATCC; Manassas, VA, USA). A549 and H1299 cells were cultured in RPMI-1640 medium (Gibco, Gaithersburg, MD, USA) supplemented with 10% fetal bovine serum (FBS, Gibco), 2 mM L-glutamine (Gibco), penicillin and streptomycin (Gibco). HEK293T cells were cultured in Dulbecco’s Modified Eagle’s Medium (Gibco) supplemented with 10% FBS, 2 mM L-glutamine, and penicillin/streptomycin. The cells were maintained in a humidified incubator under an atmosphere of 5% CO_2_ at 37 °C. All cell lines were routinely authenticated through short tandem repeat DNA profiling analyses. Cells passaged 20–30 times were used for experiments.

To generate stable HBXIP knockdown cell lines, cells were infected by lentiviral particles carrying shRNA plasmids (shHB-2: GGAACATTATGATCCAGAAAC; shHB-3: GGAACTGGATCCTACCTATGT) or control (shGFP). Similarly, stable HBXIP-overexpressing cell lines were generated by transduction with lentiviral particles containing HBXIP-FLAG or GFP cDNA. Cells were then selected by puromycin for 5–7 days after 48 h of lentiviral infection. Recombinant lentiviral particles enriched in medium supernatant were produced by co-transfection with the previously mentioned vectors and packing plasmids in HEK293T cells, and were harvested after centrifugation and filtration to remove cell debris.

### Compounds

The MEK inhibitors, PD and U0126, were purchased from Sigma-Aldrich (St. Louis, MO, USA). Compounds were dissolved in dimethyl sulfoxide (Sigma-Aldrich) and diluted with culture medium to the desired concentration for *in vitro* studies.

### Antibodies and Western blot

Cells were lysed in RIPA lysis buffer (Beyotime, Shanghai, China), and the protein concentration was measured using a BCA Protein Assay Kit (Beyotime). Protein lysates were separated by SDS-PAGE, and target proteins were detected by Western blot with the following antibodies: anti-HBXIP (Abcam, Boston, MA, USA), anti-tubulin (Huada, Shenzhen, China), anti-actin (Sigma-Aldrich), anti-Ki-67 (Abcam), anti-ERK, anti-p-ERK, MEK1, and p-MEK (Cell Signaling Technology, Danvers, MA, USA), and anti-FLAG (Sigma-Aldrich).

### Cell viability assay

Cell viability was determined by the MTS assay using a CellTiter 96® AqueousOne Solution Cell Proliferation Assay Kit (Promega, Madison, WI, USA) following the manufacturer’s instructions. Briefly, the cells were harvested from exponential phase cultures and plated in a 96-well plate at a seeding density of 5 × 10^3^ (6 wells per sample) for a specific amount of time. Subsequently, the cells were incubated at 37 °C with MTS solution for another 2–4 h. Cell survival percentage was calculated based on the absorbance of the culture mixture at 490 nm using a SpectraMax190 microplate reader (Molecular Devices, San Jose, Ca, USA).

### Colony formation assay

Cancer cells were seeded at the density of 300 cells/well in 6-well plates in duplicate and cultured for 10–12 days. Then, colonies were fixed using 4% paraformaldehyde and stained with 0.05% Crystal Violet. Macroscopic images were captured using a camera (Nikon, Tokyo, Japan).

### Flow cytometry analysis

A549 and H1299 cells were seeded in 6-well plates at approximately 40% confluence and then cultured until reaching 80%–90% confluence. The cells were then harvested and fixed with 70% ice-cold ethanol overnight. Subsequently, the cells were pelleted and resuspended in phosphate-buffered saline supplemented with 60 μg/mL RNase A and 25 μg/mL propidium iodide before being incubated for 20 min in darkness at 37 °C. The cell cycle was analyzed using a FACSCalibur flow cytometer (BD Biosciences, San Jose, CA, USA).

### Quantitative reverse transcription PCR (RT-qPCR)

Total RNA was extracted from cells using an RNeasy Mini Kit (Qiagen, Hilden, Germany). The cDNA was synthesized using a Transcriptor First Strand cDNA Synthesis Kit (Roche, Basel, Switzerland) according to the manufacturer’s instructions. PCR amplification was performed with FastStart Universal SYBR Green Master (Roche) in a iQ5 system (Bio-Rad, Hercules, CA, USA). The following PCR thermocycling conditions were used: 95 °C for 10 min followed by 40 cycles of 95 °C for 15 s, 58 °C for 15 s, and 72 °C for 30 s, after which a melting curve of the amplified DNA was acquired. Quantification of target genes was normalized using glyceraldehyde 3-phosphate dehydrogenase (GAPDH) as an internal control. The sequences of the primers used for RT-qPCR analyses were as follows: HBXIP (forward: TGCACAGATTCACAAGGACTTA and reverse: GCTGGG CTAGAACAGATATCAC); MEK1 (forward: CAATGGCGGTG TGGTGTTC and reverse: GATTGCGGGTTTGATCTCCAG); and GAPDH (forward: GGAGCGAGATCCCTCCAAAAT and reverse: GGCTGTTGTCATACTTCTCATGG).

### Immunofluorescence assay

Cells were fixed with 4% paraformaldehyde for 20 min and then permeabilized with 0.2% Triton X-100 for 30 min. After being blocked with 5% goat serum for 2 h, the cells were incubated with primary antibodies for 4–6 h at room temperature or overnight at 4 °C. Then, the cells were incubated with Alexa Fluor 488 anti-mouse or 594 anti-rabbit secondary antibodies (Molecular Probes, Eugene, OR, USA) followed by nuclei staining with Hoechst 33342 (Sigma-Aldrich) and mounting. Images were captured using a Leica TCS SP5 confocal microscope.

### Immunoprecipitation

Cells were lysed in lysis buffer [20 mM Tris-HCl (pH 8.0), 137 mM NaCl, 10% glycerol, 1% NP-40, and 2 mM EDTA] supplemented with protease inhibitors (Roche) on ice for 30 min. After centrifugation at 12,000 × *g* for 20 min, the supernatants were collected and incubated with anti-FLAG M2 magnetic beads (Sigma-Aldrich) at 4 °C overnight. The beads were then washed 3 times with lysis buffer, and the bound proteins were released from the beads by boiling the beads in 2× SDS loading buffer for 5 min. Subsequently, Western blot analysis was performed to detect the levels of proteins present in the immunoprecipitated samples.

### Tumor xenografts

BALB/c athymic nude mice were housed and treated according to the guidelines of the National Institutes of Health Guide for the Care and Use of Laboratory Animals. HBXIP knockdown (shHB-3 treated) or control (shGFP treated) A549 cells (1 × 10^7^) were subcutaneously injected into the dorsal flanks of mice (*n* = 5). Tumor volume was monitored approximately every 3 days and calculated according to the following formula: Volume (mm^3^) = 1/2 × length × width^2^. Tumors were excised at the terminal time point, processed and analyzed by IHC staining. All animal procedures were approved by the Animal Care Committee of Tianjin Medical University (approval number: AE2020124).

### Statistical analysis

Statistical analyses were performed using SPSS 12 statistical software for Windows (IBM, Armonk, NY, USA). Differences between groups were assessed by an unpaired Student’s *t*-test, nonparametric Mann-Whitney test, and chi-squared test. Survival curve analysis was performed using the Kaplan-Meier test. The Cox proportional hazards ratio method was used to assess the simultaneous effect of multiple predictors of survival. All results are presented as the mean ± SEM, and *P* values are indicated in the figures (***P < 0.001; **P < 0.01; *P* < 0.05). *P* < 0.05 was considered to be significant.

## Results

### HBXIP is upregulated in NSCLC and correlated with NSCLC progression and poor prognosis

**Figure 1:**
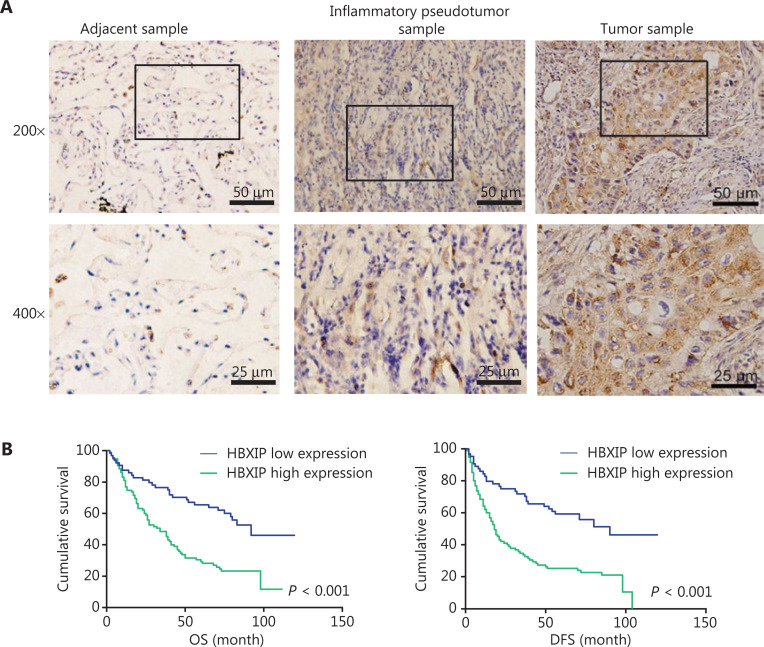
HBXIP is upregulated in non-small cell lung cancer (NSCLC) specimens and correlates with cancer progression. (A) Immunohistochemical staining of HBXIP in NSCLC samples, adjacent samples, and inflammatory pseudotumor samples. Representative images with different HBXIP staining intensities are shown. (B) Overall survival and disease-free survival were analyzed for NSCLC patients with different HBXIP expression levels. HBXIP level was scored as two grades [low (a final score < 3) and high (a final score of 4–12)] by multiplying the percentage of positive cells and the immunostaining intensity value.

To evaluate the oncogenic role of HBXIP and determine whether its expression was correlated with NSCLC clinicopathological features, 159 NSCLC samples were assayed using tissue arrays, and 40 lung adjacent samples and 40 inflammatory pseudotumor samples were also analyzed by IHC staining. Representative images of samples with different HBXIP expression levels are shown in **Figure 1A**. The correlations between HBXIP expressions and clinicopathological features of NSCLC are shown in **Table 1**. Higher HBXIP expression in NSCLC compared to that observed in matched normal thyroid tissues (**Supplementary Figure S1A**) was significantly correlated with sex (*P* = 0.033), N stage (*P* = 0.002), and tumor-node-metastasis (TNM) stage (*P* = 0.008) but was independent of other factors, including age, smoking, type of resection, histological subtype, and T stage (**Table 1**). All slides were reviewed by two pathologists in a double-blinded manner for IHC scoring based on the percentages and intensities of HBXIP staining signals. In addition, Western blot results further confirmed the increased expression of HBXIP in NSCLC samples (**Supplementary Figure S1A**). To further support our results, we also analyzed samples from the Gene Expression Omnibus and The Cancer Genome Atlas RNA-Seq database. The results showed that HBXIP expression in NSCLC was significantly higher than that observed in matched normal tissues, and was also positively associated with NSCLC progression (**Supplementary Figure S1B**). We further examined the effects of HBXIP expression levels on the survival of lung cancer patients. The results showed that the 6-year overall survival and disease-free survival (DFS) of the HBXIP high expression group were significantly lower than those of the low expression group (*P* < 0.001 and *P* < 0.001, respectively) (**Figure 1B** and **Table 2**). Multiple logistic regression analysis results identified the TNM stage (*P* < 0.001 and *P* < 0.001, respectively), and HBXIP expression (*P* = 0.016 and *P* = 0.006, respectively) as independent prognostic factors for OS and DFS (**Table 3**). Taken together, our findings suggested that HBXIP expression was increased in NSCLC and that its expression level was associated with NSCLC progression.

**Table 1:** Correlation of HBXIP expression and clinicopathological characteristics of NSCLC patients

Variables	Low expression	High expression	χ^2^	*P*
Gender				
Female	14	36	4.552	0.033
Male	50	59		
Age (years)				
< 60	31	41	0.430	0.512
≥ 60	33	54		
Smoking status				
Never smoke	18	30	0.216	0.642
Smoke	46	65		
Type of resection				
Pneumonectomy	9	13	0.005	0.998
Lobectomy	53	79		
Bronchial sleeve resection	2	3		
Lesion				
Central	20	28	0.057	0.811
Peripheral	44	67		
Histologic subtype				
Squamous cell carcinoma	40	62	0.127	0.722
Adenocarcinoma	24	33		
Location of tumor				
Left	26	39	0.003	0.957
Right	38	56		
Stage				
I	25	18	9.635	0.008
II	21	31		
III	18	64		
T stage				
T1	15	27	0.710	0.701
T2	41	59		
T3	8	9		
N stage				
N0	39	31	12.950	0.002
N1	10	20		
N2	15	44		

**Table 2:** Overall survival (OS) and disease-free survival (DFS) univariate analysis according to clinicopathological factors in 159 NSCLC patients

Variable	No. of patients	Percent (%)	6-Year OS rate (%)	*P*	6-Year DFS rate (%)	*P*
Gender						
Female	50	31.4	24.0	0.008	20.0	0.003
Male	109	68.6	41.3		38.5	
Age (years)						
< 60	72	45.3	38.9	0.414	36.1	0.558
≥ 60	87	54.7	33.3		29.9	
Smoking status						
Never smoke	48	30.2	27.1	0.081	25.0	0.125
Smoke	111	69.8	39.6		36.0	
Type of resection						
Pneumonectomy	22	13.8	27.3	0.076	27.3	0.151
Lobectomy	132	83.0	36.4		32.6	
Bronchial sleeve resection	5	3.1	60.0		60	
Lesion						
Peripheral	111	69.8	35.1	0.503	32.4	0.415
Central	48	30.2	37.5		33.3	
Histologic subtyp						
Squamous cell carcinoma	102	64.2	37.3	0.886	34.3	0.871
Adenocarcinoma	57	35.8	33.3		29.8	
Location of tumor						
Left	65	40.9	33.8	0.420	27.7	0.203
Right	94	59.1	37.2		36.2	
Stage						
I	43	27.0	60.5	<0.001	53.5	<0.001
II	52	32.7	44.2		40.4	
III	64	40.3	12.5		12.5	
HBXIP expression						
Low	64	40.3	54.7	<0.001	51.6	<0.001
High	95	59.7	23.2		20.0	

**Table 3:** Overall survival and disease-free survival multivariate analysis according to clinicopathological factors in 159 NSCLC patients

Variables	Overall survival		Disease-free survival	
	HR (95% CI)	*P*	HR (95% CI)	*P*
Female	1.308 (0.858–1.994)	0.212	1.355 (0.896–2.047)	0.150
TNM stage	2.021 (1.52–2.682)	<0.001	1.947 (1.481–2.558)	<0.001
HBXIP expression	1.763 (1.114–2.793)	0.016	1.865 (1.198–2.904)	0.006

HR, hazard ratio; CI, confidence interval; TNM, tumor/node/metastasis.

### HBXIP promotes NSCLC progression *in vitro*

**Figure 2:**
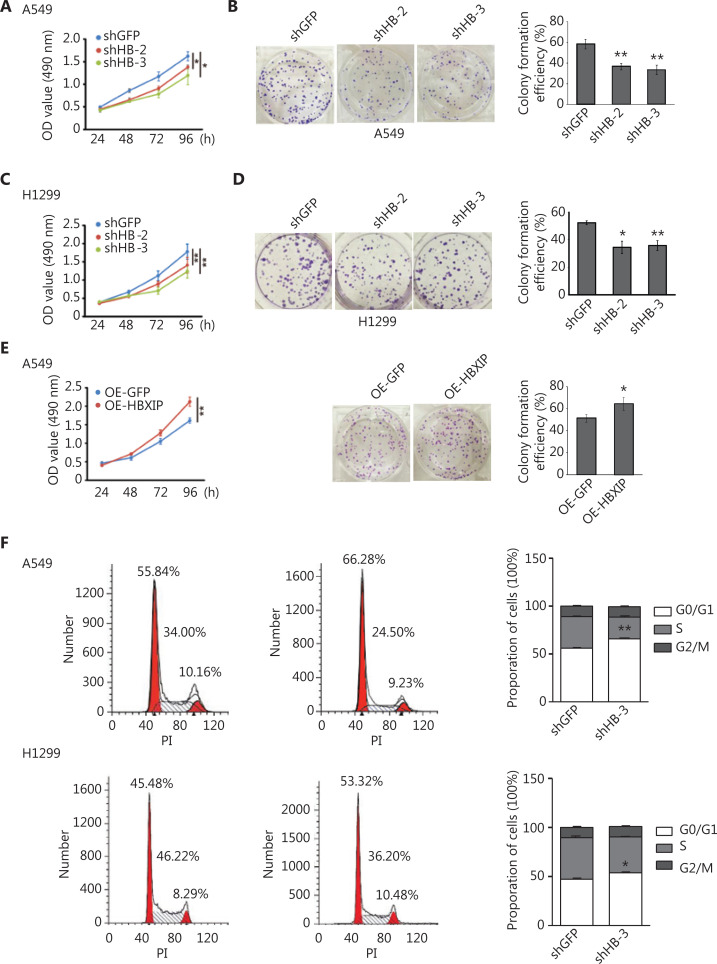
HBXIP promotes non-small cell lung cancer progression *in vitro*. (A) and (C) Cancer cell viability was examined using the MTS assay in HBXIP stable knockdown cells and control cells. (B) and (D) The colony formation assay was performed in HBXIP stably knocked down cells. A549 and H1299 cells were cultured for 12 days prior to Crystal Violet staining. (E) Left panel; the viability of HBXIP-overexpressing A549 cells was examined using the MTS assay. Right panel; representative images of forming colonies and their estimated numbers. HBXIP-overexpressing and control A549 cells were fixed and stained 12 days after seeding. (F) Cell cycle analysis of HBXIP-deficient cells. The numbers of viable A549 and H1299 cells in the G0/G1, S, and G2/M phases were individually quantified by flow cytometry. Representative profiles (left panel) and the corresponding percentages of each stage are shown. The results were analyzed by an unpaired *t*-test. *P < 0.05; **P < 0.01.

The role of HBXIP in NSCLC tumorigenesis was investigated *in vitro* (**Supplementary Figure S1C**) using two NSCLC cell lines (A549 and H1299), and HBXIP was either stably knocked down or overexpressed in these cell lines to conduct functional studies. The knockdown efficiency of HBXIP was confirmed at the mRNA and protein levels by RT-qPCR and Western blot, respectively (**Supplementary Figure S2A, S2B**). The effect of HBXIP on NSCLC cell growth was assessed using a MTS assay, which showed that HBXIP knockdown significantly decreased the growth of both the A549 and H1299 cell lines (**Figure 2A, 2C**). Consistent with these results, HBXIP knockdown decreased the colony formation ability of NSCLC cells (**Figure 2B, 2D**). Next, we stably overexpressed HBXIP in A549 cells (**Supplementary Figure S2C**), which was validated by Western blot analysis. The MTS and colony formation assay results showed that HBXIP overexpression promoted the proliferation and colony formation ability of A549 cells (**Figure 2E**). Furthermore, the wound healing assay results showed that HBXIP knockdown in A549 and H1299 cells abrogated their migration, when compared to that of the controls (**Supplementary Figure S2D**).

The ability of HBXIP to inhibit NSCLC cell proliferation through cell cycle arrest was assessed by flow cytometry [fluorescence-activated cell sorting (FACS)] analysis. The data showed that HBXIP knockdown concomitantly increased the population of G1 phase cells and decreased that of S phase cells, when compared with that in the control group (**Figure 2F**), which was indicative of cell cycle arrest. Collectively, these results indicated that HBXIP enhanced the proliferative capacity and cell cycle progression of NSCLC cells.

### HBXIP-mediated promotion of NSCLC cancer progression is dependent on the MAPK/ERK pathway

**Figure 3:**
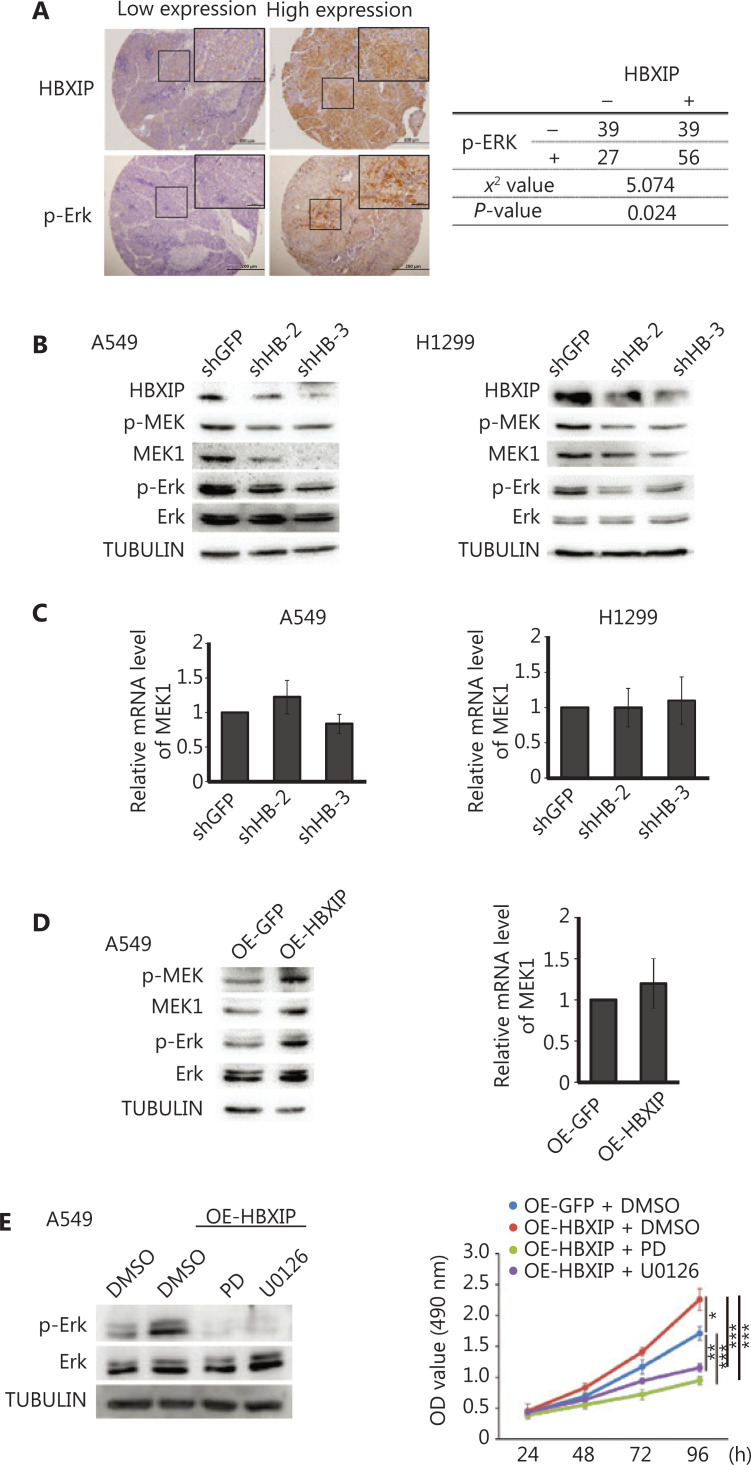
HBXIP promotes non-small cell lung cancer progression through the MAPK/ERK pathway. (A) Phosphorylated ERK levels in the NSCLC cohort samples were analyzed by immunohistochemical staining, and the correlation between p-ERK and HBXIP was analyzed. (B) Phosphorylated MEK and ERK levels in HBXIP-deficient A549 and H1299 cells were analyzed by Western blot. (C) MEK1 mRNA levels in HBXIP knockdown A549 and H1299 cells were examined by RT-qPCR. (D) Western blots of phosphorylated MEK and ERK levels (left panel) and MEK1 mRNA levels in HBXIP-overexpressing A549 cells. (E) Phospho-ERK levels in HBXIP-overexpressing A549 cells treated with PD (10 μM) or U0126 (10 μM) for 24 h were determined by Western blot analysis. (E) MTS assay measuring the viability of HBXIP-overexpressing A549 cells treated with PD (10 μM) or U0126 (10 μM) for the indicated times. The results were analyzed by an unpaired *t*-test. *P < 0.05; **P < 0.01; and ***P < 0.001.

HBXIP, also termed LAMTOR5, is a member of the late endosomal or lysosomal adaptor and MAPK and mTOR activator (LAMTOR) family^23,25^. The MAPK/ERK signal pathway is well-established as one of the most important pathways in NSCLC, stimulating our interest in whether HBXIP could promote NSCLC development through the MAPK/ERK pathway. IHC staining was performed using the NSCLC cohort samples to assess p-ERK expression levels, the results of which showed a positive correlation between HBXIP expression and p-ERK activation (**Figure 3A**). Subsequently, Western blot analysis of NSCLC cell lines with stable HBXIP knockdown was performed, the results of which showed that HBXIP deficiency significantly decreased the phosphorylation levels of ERK1/2 proteins. Notably, the total protein levels of MEK1, a key member of the MAPK/ERK pathway, were also reduced in the two NSCLC cell lines as a result of HBXIP deficiency (**Figure 3B**). This reduction in MEK1 protein levels was not due to transcriptional regulation, as indicated by unaltered mRNA expression levels (**Figure 3C**). Taken together, these results indicated that HBXIP regulated MEK1 at the protein level, but not at the transcriptional level.

Next, we performed experiments to confirm the HBXIP-mediated regulation of MEK1 protein levels using A549 cells stably overexpressing HBXIP. Western blot results showed that HBXIP overexpression significantly increased both the phosphorylated and total MEK1 levels as well as downstream phosphorylated ERK1/2 levels, suggesting that HBXIP activated the MAPK/ERK pathway (**Figure 3D**). Moreover, the mRNA levels of MEK1 were not affected by HBXIP overexpression (**Figure 3D**). To further demonstrate that MEK1 played an important role in the HBXIP-mediated promotion of NSCLC progression, we assessed phosphorylated ERK1/2 protein levels in A549 cells stably overexpressing HBXIP treated with the MEK inhibitors, PD and U0126. The results showed that activation of the MAPK/ERK pathway by HBXIP overexpression was abolished by treatment with the PD and U0126 inhibitors (**Figure 3E**). Next, these inhibitors were assessed for their abilities to affect cell viability in HBXIP-overexpressing A549 cells. As expected, PD and U0126 treatment completely abrogated the cell growth advantage conferred by HBXIP overexpression, reducing cell growth rates to even lower than that observed in the DMSO control group (**Figure 3E**). Collectively, these results showed that HBXIP affected the MAPK/ERK pathway by regulating MEK1 protein levels, but not the mRNA levels, potentially explaining how HBXIP-mediated promotion of NSCLC development was partially dependent on the MAPK/ERK pathway.

### HBXIP interaction with MEK1 stabilizes its protein level

**Figure 4:**
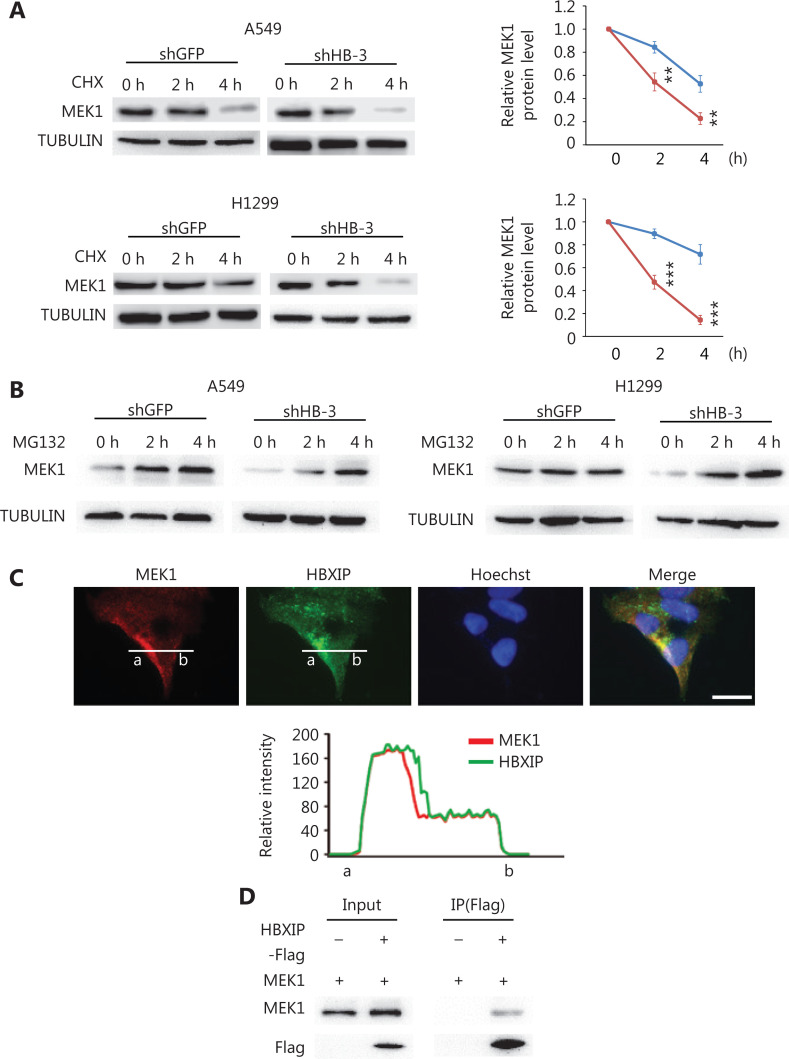
HBXIP prevents proteasome-mediated MEK1 degradation. (A) MEK1 protein stability in HBXIP-deficient A549 and H1299 cells was assessed by Western blot (left panel), and protein levels were quantified (right panel) after cells were treated with cycloheximide for 2 or 4 h. (B) Western blot of MEK1 protein in HBXIP knockdown A549 and H1299 cells treated with MG132 for 2 and 4 h. (C) Immunofluorescence staining of HBXIP and MEK1 in HBXIP-overexpressing A549 cells and the quantified staining intensity of HBXIP and MEK1 (lower panel). Scale bars, 50 μm. (D) Immunoprecipitation of MEK1 by anti-FLAG beads in cell extracts of A549 cells overexpressing FLAG-tagged HBXIP. The results were analyzed by an unpaired *t*-test. **P < 0.01; ***P < 0.001.

The observed HBXIP-mediated regulation of MEK1 protein levels suggested that HBXIP may alter MEK protein stability. To test this possibility, we measured the degradation rate of MEK1 protein under the inhibition of protein synthesis by cycloheximide. HBXIP protein was significantly degraded after a 4 h cycloheximide treatment upon HBXIP knockdown, when compared to that observed in the GFP controls (**Figure 4A**). These results indicated that HBXIP knockdown promoted the degradation of MEK1. Next, we examined whether MEK1 degradation upon HBXIP knockdown depended on the proteasome pathway. To this end, the proteasome inhibitor MG132 was used to treat the HBXIP knockdown and controls of two distinct NSCLC cell lines. The results showed that the reduction in MEK protein levels in HBXIP knockdown cells was completely reverted upon MG132 treatment, normalizing to that observed in the controls, and suggesting that a proteasome-dependent mechanism contributed to the degradation of MEK1 (**Figure 4B**). Importantly, confocal microscopy showed co-localization of HBXIP and MEK1 in A549 cells (**Figure 4C**). Moreover, co-immunoprecipitation assay results showed that MEK1 was precipitated by FLAG-tagged HBXIP in A549 cells using anti-FLAG antibody (**Figure 4D**). However, attempts to precipitate HBXIP using FLAG-tagged MEK1 were unsuccessful (data not shown), possibly because endogenous MEK1 levels were already high, causing the majority of HBXIP to bind endogenous MEK2 rather than ectopic flag-MEK1. Taken together, these results indicated that HBXIP interacted with MEK1 and prevented its proteasomal degradation.

### HBXIP knockdown inhibits NSCLC progression *in vivo*

**Figure 5:**
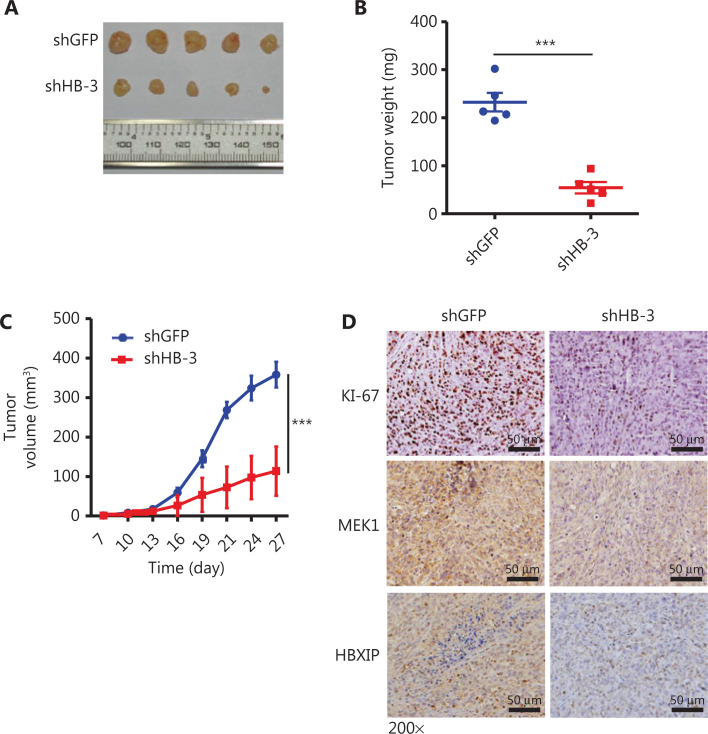
HBXIP knockdown inhibits non-small cell lung cancer development *in vivo*. (A)–(C) Xenografts of HBXIP knockdown (shHB-3) and control (shGFP) A549 cells in athymic mice. Macroscopic images (A) and weights (B) of tumors dissected from mice on day 27 (*N* = 5). Tumor volumes (C) were measured every 3 days. (D) Immunohistochemical staining of Ki-67, MEK1, and HBXIP in tumor sections from mice described in (A). The results were analyzed by an unpaired *t*-test. ***P < 0.001.

The function of HBXIP in NSCLC tumorigenesis was further investigated *in vivo* using a xenograft model in which HBXIP knockdown or control A549 cells were subcutaneously implanted into nude mice. The results showed that HBXIP knockdown caused a dramatic reduction in both tumor weight (**Figure 5A, 5B**) and volume (**Figure 5C**). Importantly, the IHC staining results showed that HBXIP knockdown tumors had a low percentage of Ki-67 positive cells, indicative of reduced proliferation. In agreement with this result, decreased expression levels of MEK1 were also observed in HBXIP knockdown tumors (**Figure 5D**). Taken together, these results suggested an important role of HBXIP in NSCLC tumor growth *in vivo*.

## Discussion

The MAPK/ERK pathway is essential in the regulation of cell proliferation and survival in human tumorigenesis^26–29^. The importance of this pathway has been well-established in lung cancer development, particularly for NSCLC^30^. In lung cancer, the MAPK pathway is driven by oncogenic mutations (e.g., *BRAF* and *RAS* mutations), *RET-PTC,* and in some cases by the recently discovered *ALK* mutations^31–33^. MEK1 is an important member of the MEK family that is commonly responsible for MAPK/ERK pathway activation^34,35^. Thus, it is conceivable that upregulation in MEK1 protein levels may contribute to the initiation, progression, and therapeutic response of tumors.

HBXIP has been identified as an oncogene, which participates in crucial biological processes in a broad variety of cancer types. Nevertheless, its role in NSCLC has remained unclear. In the present study, for the first time, we provided evidence that HBXIP may have an oncogenic role in NSCLC development. IHC staining, Western blot, and RT-qPCR data showed high HBXIP expression was frequently detected in NSCLC specimens and was positively correlated with advanced tumor grade, highlighting its potential biological roles in the development and progression of NSCLC.

During liver and breast cancer development, HBXIP reportedly promotes tumor cell proliferation through transcriptional activation of crucial tumor oncogenes, including YAP, SKP2, and Lin28B^13,19,21^. In the present study, we observed that HBXIP knockdown in NSCLC cells resulted in an accumulation of G1 phase cells and a consequential decrease in proliferation both *in vitro* and *in vivo*. Notably, aggressive NSCLC phenotypes mediated by HBXIP are at least partially dependent on the MAPK/ERK pathway. Next, we sought to identify the mechanism by which HBXIP activates this pathway in NSCLC cancer cells. The results showed that HBXIP upregulated the protein levels, but not mRNA levels, of MEK1, indicating that HBXIP activated the MAPK/ERK pathway through a transcription-independent mechanism. Subsequently, the mechanism associated with this activity was shown to involve the HBXIP-mediated stabilization of MEK1 protein, which prevented its proteasome-dependent degradation.

Several studies have reported that HBXIP activates the MAPK/ERK pathway through different mechanisms. Cui et al.^36^ showed that HBXIP upregulates CD46, CD55, and CD59 by activating the ERK1/2 signaling pathway to protect breast cancer cells from complement attack. However, the potential mechanism by which HBXIP upregulates ERK1/2 phosphorylation levels has not been elucidated. In another study, Li et al.^37^ demonstrated that HBXIP sequentially activated MEKK2/ ERK1/2/Canp4 signaling, leading to an enhanced migration of breast cancer cells. Moreover, chromatin immunoprecipitation (ChIP) assay results showed that HBXIP bound to the MEK2 promoter, but not that of MEK1 or MEK3 in breast cancer cells, suggesting that HBXIP may activate ERK1/2 by promoting MEK2 expression. In addition, HBXIP/LAMTOR5 is a membrane protein specifically localized to the surface of late endosomes/lysosomes, which serves as an anchor for the “Ragulator” complex, comprised of p14/LAMTOR2, MP1/LAMTOR3, p18/LAMTOR1, and C7orf59. The ragulator complex can also regulate a branch of the MAPK pathway by recruiting MEK1 to late endosomes/lysosomes^38,39^. In the present study, we provided new insights into the HBXIP-mediated regulation of the MAPK/ERK pathway. Our results showed that HBXIP stabilized MEK1 protein and thereby activated the MAPK/ERK pathway, which contributed to NSCLC development. Moreover, treatment with an MEK inhibitor abolished the growth advantage of NSCLC cells resulting from HBXIP overexpression. This observation may be of important clinical relevance, because HBXIP overexpression is frequently observed in tumor samples from NSCLC patients, and may confer tumor resistance to MEK inhibitors, which are broadly prescribed as clinical drugs to treat NSCLC. Taken together, our results showed that HBXIP-overexpressing NSCLC cells activated MAPK/ERK signaling, indicating that HBXIP may be a potential therapeutic target for NSCLC treatment.

Given the complexity of molecular signaling pathways, the precise mechanism by which HBXIP stabilizes MEK1 for subsequent activation of MAPK/ERK signaling was not elucidated in the present study. Considering that HBXIP functions as a part of the LAMTPOR complex, whether the latter component is implicated in the interaction of HBXIP with MEK1 requires further investigation. The results of previous studies indicated that the MP1 complex, another member of the LAMTPOR family, formed a complex with p14 as a scaffold for MEK1 recruitment into the endosomal compartment, which is essential for MER/ERK activity^38–40^. Furthermore, these previous studies showed that the MP1 directly bound to MEK1, which partly explained why HBXIP could not be co-immunoprecipitated by FLAG-MEK1. Moreover, because the LAMTOR2/LAMTOR3 heterodimer serves as a scaffold for MEK1 on late endosomes to activate MEK/ERK signaling pathway^23,40–42^, it is possible that the interaction between HBXIP (also termed LAMTOR5) and MEK1 relied on the formation of the LAMTOR complex comprising LAMTOR2, LAMTOR3 and HBXIP. Based on these observations, it is conceivable that the mechanism by which HBXIP binding to MEK1 activates MAPK/ERK signaling involved HBXIP either functioning as a coactivator that directly increased MEK/ERK activity or alternatively stabilized MEK1 to promote MAPK/ERK signaling. Another remaining question is how HBXIP stabilizes the MEK1 protein, a process that may involve the ubiquitination of MEK1, which is presumably blocked by interaction with the LAMTOR complex. In addition, another limitation of the present study involves the inclusion of clinical case samples. The NSCLC cohort consisted of more cases of squamous cell carcinomas than adenocarcinomas, which may not reflect the conventional histological type distribution of NSCLC. Thus, additional investigations should involve the use of more lung adenocarcinoma samples with an NSCLC detection panel to confirm our conclusions. However, we still obtained meaningful results using the defective detection panel, which indicated that high expression of HBXIP in NSCLC was significantly correlated with sex, N stage, and TNM stage, and identified HBXIP expression as an independent prognostic factor of overall survival and disease-free survival. Furthermore, these results contributed to the continued development of the optimal NSCLC detection panel.

## Conclusions

**Figure 6:**
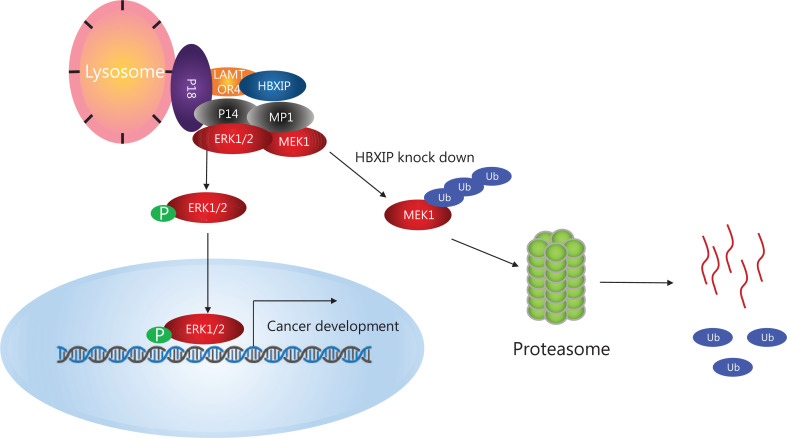
Schematic model of the role of HBXIP in regulating non-small cell lung cancer (NSCLC). HBXIP promotes NSCLC cell proliferation and tumorigenesis, possibly by activating the MAPK/ERK pathway, and HBXIP stabilizes MEK1 and prevents its degradation by the proteasome pathway.

In summary, for the first time, the results of our present study showed that HBXIP played an essential role in NSCLC progression. HBXIP was able to promote NSCLC cell proliferation and tumorigenesis, possibly by activating the MAPK/ERK pathway. We identified MEK1 as a novel downstream target of HBXIP and showed that HBXIP stabilized MEK1 and prevented its degradation through the proteasome pathway. These results will advance our understanding of the oncogenic role of HBXIP, and indicates its potential as a novel diagnostic biomarker in NSCLC and as a therapeutic target in combination with MAPK inhibitors in cancer treatment (**Figure 6**).
