# Supplementary material for: Oncoprotein HBXIP promotes tumorigenesis through MAPK/ERK pathway activation in non-small cell lung cancer
Source: Cancer Biol Med. 2021 Feb 15;18(1):105–19. doi: 10.20892/j.issn.2095-3941.2020.0098 (PMC7877173; doi:10.20892/j.issn.2095-3941.2020.0098)
Supplement: Supplementary file 1 [file cbm-18-105-s001.pdf]

Supplementary materials

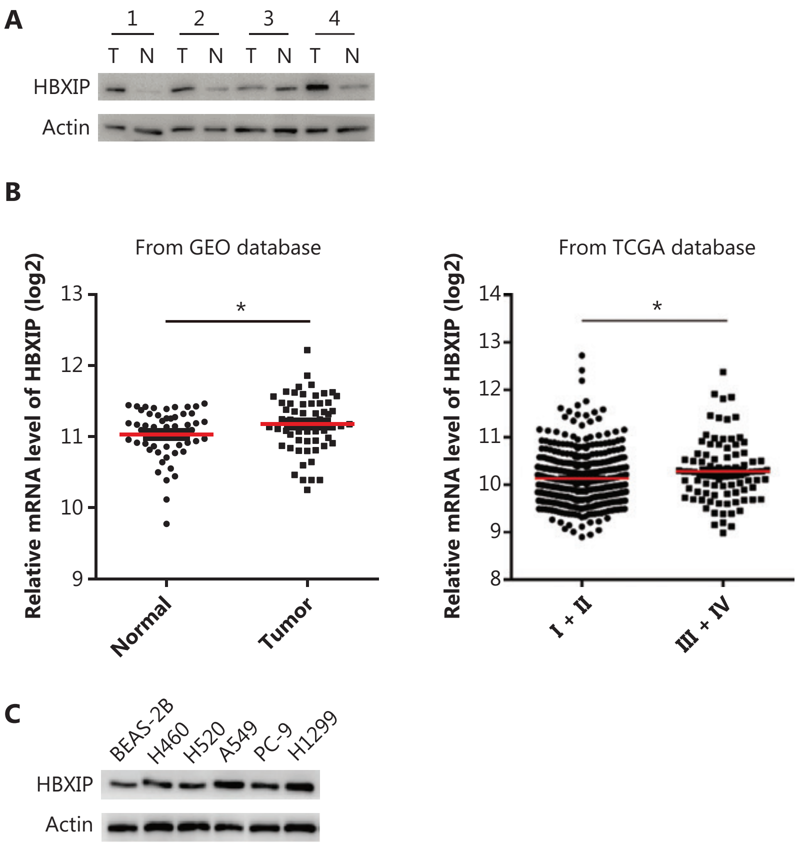

Figure S1 HBXIP is upregulated in NSCLC specimens and correlated with cancer progression. A. Western blot showing that the expression levels of HBXIP expression in 3 out of 4 NSCLC specimens (denoted as T) were higher than that observed in their matched normal thyroid tissues (denoted as N). B. Analysis of HBXIP mRNA levels in NSCLC samples and normal lung tissues were analyzed by from the GEO database (left panel). Correlation of HBXIP mRNA levels with TNM stage in NSCLC samples were from the TCGA database (right panel). Results The results were analyzed by Mann WhitneyMann-Whitney U test. C. Western blot results showing that the protein expression the level of HBXIP protein expression in six different lung cancer cell lines.

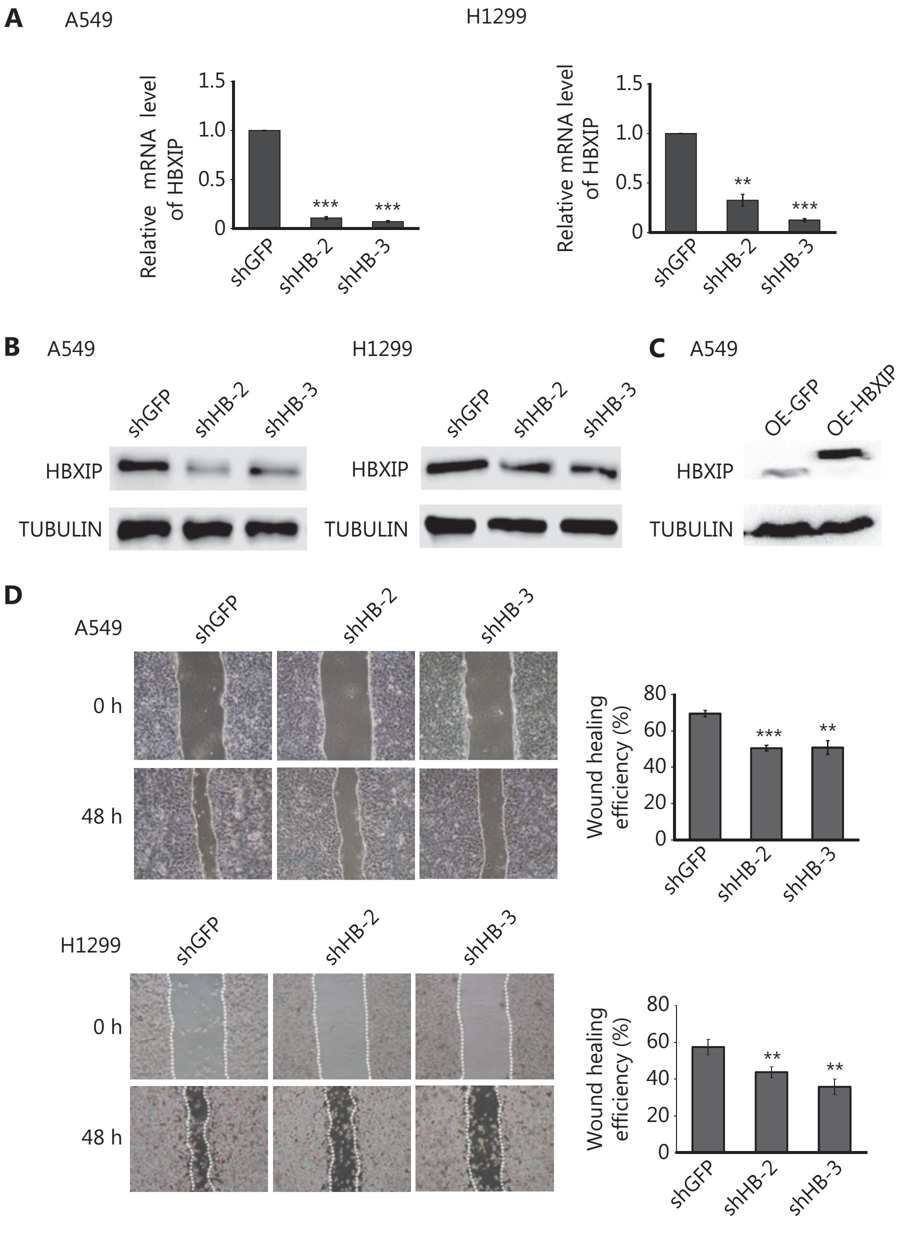

Figure S2 HBXIP knockdown reduced the migration. AB. The knockdown Knockdown efficiency of HBXIP by lentivirus-delivering in A549 and H1299 cells by lentiviral delivery of HBXIP-specific shRNA (shHB-2 and shHB-3) in A549 and H1299 cells was as detected by RT-qPCR and Western blot analysis, where shGFP serves served as a control. BC. Western blot of HBXIP protein levels in A549 cells overexpressing HBXIP compared to that observed in control cells. CD. Representative images of wound healing assays showing that knockdown of HBXIP knockdown reduces the migration capacity in of A549 and H1299 cells. Right The right panels showing show the quantification of the wound healing efficiency. Results The results were analyzed by unpaired t-test. ***P* < 0.01, *** *P* < 0.001.
